# C_2_-Ceramide-Induced Rb-Dominant Senescence-Like Phenotype Leads to Human Breast Cancer MCF-7 Escape from *p*53-Dependent Cell Death

**DOI:** 10.3390/ijms20174292

**Published:** 2019-09-02

**Authors:** Wen-Tsan Chang, Chang-Yi Wu, Yin-Chieh Lin, Min-Tsui Wu, Kai-Li Su, Shyng-Shiou Yuan, Hui-Min David Wang, Yao Fong, Yi-Hsiung Lin, Chien-Chih Chiu

**Affiliations:** 1 Division of Hepatobiliary and Pancreatic Surgery, Department of Surgery, Kaohsiung Medical University Hospital, Kaohsiung 807, Taiwan; 2 Department of Surgery, School of Medicine, College of Medicine, Kaohsiung Medical University, Kaohsiung 807, Taiwan; 3 Department of Biological Sciences, National Sun Yat-Sen University, Kaohsiung 804, Taiwan; 4 Department of Biotechnology, Kaohsiung Medical University, Kaohsiung 807, Taiwan; 5 Translational Research Center, Cancer Center, Department of Medical Research, Department of Obstetrics and Gynecology, Kaohsiung Medical University Hospital, Kaohsiung Medical University, Kaohsiung 807, Taiwan; 6 Graduate Institute of Biomedical Engineering, National Chung Hsing University, Taichung 402, Taiwan; 7 Chest Surgery, Chi-Mei Medical Center, Yung Kang City, Tainan 901, Taiwan; 8 Department of Physiology, College of Medicine, Kaohsiung Medical University, Kaohsiung 807, Taiwan; 9 Graduate Institute of Medicine, College of Medicine, Kaohsiung Medical University, Kaohsiung 807, Taiwan; 10 Center for Stem Cell Research, Kaohsiung Medical University, Kaohsiung 807, Taiwan; 11 Translational Research Center and Department of Medical Research, Kaohsiung Medical University Hospital, Kaohsiung 807, Taiwan

**Keywords:** breast cancer, apoptosis, senescence-like phenotype, C_2_-ceramide

## Abstract

Ceramide is a sphingolipid which regulates a variety of signaling pathways in eukaryotic cells. Exogenous ceramide has been shown to induce cellular apoptosis. In this study, we observed that exogenous ceramide induced two distinct morphologies of cell fate following C_2_-ceramide treatment between the two breast cancer cell lines MCF-7 (wild type *p*53) and MDA-MB-231 (mutant *p*53) cells. The growth assessment showed that C_2_-ceramide caused significant growth inhibition and apoptosis in MDA-MB-231 cells through down-regulating the expression of mutant *p*53 whereas up-regulating the expression of pro-apoptotic Bad, and the proteolytic activation of caspase-3. However, senescence-associated (SA)-β-galactosidase (β-gal) was regulated in MCF-7 cells after C_2_-ceramide treatment. The results of proliferation and apoptosis assays showed that MCF-7 cells were more resistant to C_2_-ceramide treatment compared to MDA-MB-231 cells. Furthermore, C_2_-ceramide treatment induced a time-responsive increase in Rb protein, a key regulator of senescence accompanied with the upregulation of both mRNA level and protein level of SA-genes PAI-1 and TGaseII in MCF-7 but not in MDA-MB-231 cells, suggesting that some cancer cells escape apoptosis through modulating senescence-like phenotype. The results of our present study depicted the mechanism of C_2_-ceramide-resistant breast cancer cells, which might benefit the strategic development of ceramide-based chemotherapeutics against cancer in the future.

## 1. Introduction

Breast cancer is the major cause of female cancer deaths worldwide, with the highest mortality and poor prognosis. It is expected to accumulate over 30% of 880,000 new cancer cases as reported by the American Cancer Society (ACS) in 2018 and it causes more than 70,000 deaths than any other cancer. To date, breast cancer remains a leading cause of public health issues for women and the most common tumor for the past two decades [1].

Senescent (aging) cells lose their ability to divide because of the limited quota of divisions [2]. Senescent cells exert the arrest of proliferation and acidic β-galactosidase (β-gal) activity (pH 6.0) with enlarged and flattened morphology [2]. In contrast, senescence-like phenotype is a growth arrest that could be induced by stresses including hypoxic stress [3], accumulation of endogenous reactive oxygen species (ROS), and some anti-tumor agents. In comparison with replicative cells, stress-induced senescence/senescent-like phenotype might occur within weeks without telomere-shortening [4].

Various signaling transductions are found to be associated with cellular senescence. Direct evidence of limited telomere cell presents a DNA damage-like response and activates the signaling regarding growth arrest and senescent status [5], with the up-regulation of cell cycle inhibitor *p*21. *p*21 deletion rescued the intestinal progenitor cell proliferation and improved the self-renewal capacity of hematopoietic stem cells; moreover, the signaling pathway, including ATM and ATR, resulted in down-stream H2AX phosphorylation, and Chk1 and Chk2 in *p*53 modification and *p*21/*p*16 activation all participate in the cell cycle-arrest effect of senescence [6]. Furthermore, the oncogene-induced senescence in response to the oncogene activation or the loss of tumor suppresses regulation to protect cell integrity and restrain cell growth [7]. Early evidence of over-expressed oncogene RAS resulted in cell cycle arrest with the rise of *p*53 and *p*16 activity, and other reports reveal that the RB pathway contributes to the senescence-induced tumor cell death [8,9]. Since then, more and more regulators are reported as being involved in senescence-initiating responses such as BRAF mutation, c-Myc suppression, ATM pathway-mediated disturbance of cdc6, and Cyclin E activation [3,10,11].

However, another accumulated report reveals the reverse role of senescence for the rare escape of tumor cells from drug-induced cell death exhibiting unstable multiplication characteristics. Some evidence suggests that these tumor cells harbor stem cell-like phenotype with CD44^+^/CD24^−^ marker features [12]. Most importantly, it is found that there are side population cells showing more resistance to antitumor agent treatment due to the enrichment of drug efflux transporter protein expression and the efficient capacity of DNA repair [13,14]. After the drug treatment, the cell death-resistant cells are identified with an appearance of the senescent population and stem cell-marker enrichment features.

*p*53 is a nuclear transcription factor with tumor-suppressing biological function in the cellular process, showing pro-apoptosis characteristics. Normally, *p*53 is activated to induce its pro-apoptosis function regarding cell cycle arrest, DNA repair, and initiation of apoptosis-related signaling [15,16,17]. However, over 50% of human cancers exhibit the loss of *p*53 with genomic loci mutation, especially in solid tumors. Accumulative evidence shows that the mutant *p*53 acts as a dominant-negative inhibitor toward the wild-type *p*53 pathway in various cancers, suggesting that cancer cells harboring mutant *p53* exhibit chemo-resistant potential and become more malignant [18,19,20]. 

A future therapeutic target for the approach in tumors with *p*53 mutations appears to be an important way to improve drug treatment outcomes. For example, a small compound NSC59984 screened from National Cancer Institute (NCI; Bethesda, MD) induces the degradation of mutant *p*53 protein in human colon cancer cells SW480 via MDM2 and the ubiquitin-proteasome pathway [21]. Another study also showed that arsenic trioxide degrades mutant *p*53 in HaCaT colon cancer cells, SW480 cells, and MIA PaCa-2 cells, with the authors finding that arsenic trioxide caused decreased stability of the mutant *p*53 protein through a proteasomal pathway in HaCaT cells [22].

Chemoresistance to cancer therapeutics remains an important problem regarding treatment failure and needs to be addressed in order to reduce cancer death rate; nevertheless, a clarification of the key mechanism and biological function related to chemoresistance will always remain important for cancer therapeutics. Senescence as an alternative cellular process shows its crucial role of an important cell self-protection mechanism against internal/external pressures [23] and suggests that cellular non-autonomous senescence responses may be partially involved in chemoresistance during treatment.

Ceramides are sphingolipids of the cell membrane that serve as a mediator of sphingolipid metabolism and many fundamental cellular pathways [24]. It has also been reported to serve as a tumor suppressor lipid against the growth of tumor cells [25,26,27,28]. For example, the effects of short carbon chain C_2_-ceramide on apoptosis in lung cancer cells have been studied previously [27,28]. Ceramide has been shown to exert strong potential for regulating cell apoptosis, cell cycle arrest, and autophagic responses [29,30]. Our previous studies have demonstrated the potential effects of ceramides in anticancer by regulating various cellular pathways. To investigate the effect of C_2_-ceramide in inducing cell senescence and its association with *p*53 status, two different types of breast cancer cells, harbored wild-type *p*53 (MCF-7) and mutant *p*53 (MDA-MB231), were used in this study to further clarify the cell response of apoptosis and chemoresistance toward C_2_-ceramide.

## 2. Results

### 2.1. Discrepant Anti-Proliferation Effects of C_2_-Ceramide on Two Breast Cancer Cells

**Figure 1:**
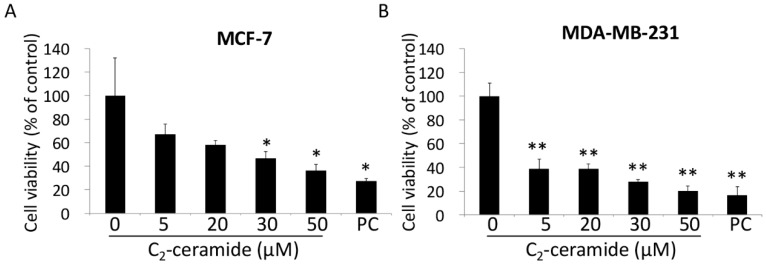
The inhibitory effect of C_2_-ceramide on breast cancer cells. Two breast cancer cell lines MCF-7 (**A**) and MDA-MB-231 (**B**) were treated with the indicated concentrations (from 5 to 50 μM) of C_2_-ceramide for 24 h respectively. The cell proliferation was determined using the PreMix WST-1 assay. Nought indicates the cells were treated with C_2_-ceramide-free solvent as vehicle control. Positive Control (PC): 0.5 μM 2,9-Bis [2-(pyrrolidin-1-yl) ethoxy]-6-{4-[2-(pyrrolidin-1-yl) ethoxy] phenyl}-11H-indeno [1,2-c] quinolin-11-one (BPIQ), a camptothecin analog. * *p* < 0.05 and ** *p* < 0.001 for C_2_-ceramide versus control respectively.

Samples from two breast cancer cell lines MCF-7 and MDA-MB-231 were treated with indicated concentrations of C_2_-ceramide for 24 h respectively, and the rates of cellular proliferation were determined. The results of cellular proliferation assay showed that the inhibitory effect of C_2_-ceramide on the proliferation of the two breast cancer cell lines is discrepant (Figure 1). The IC_50_ measures of C_2_-ceramide in MCF-7 and MDA-MB-231 were 27.13 µM and four µM respectively. This suggests that MDA-MB-231 was more sensitive to C_2_-ceramide compared to MCF-7. 

### 2.2. C_2_-ceramide Induced Senescence-Like Phenotype of MCF-7 Cells

**Figure 2:**
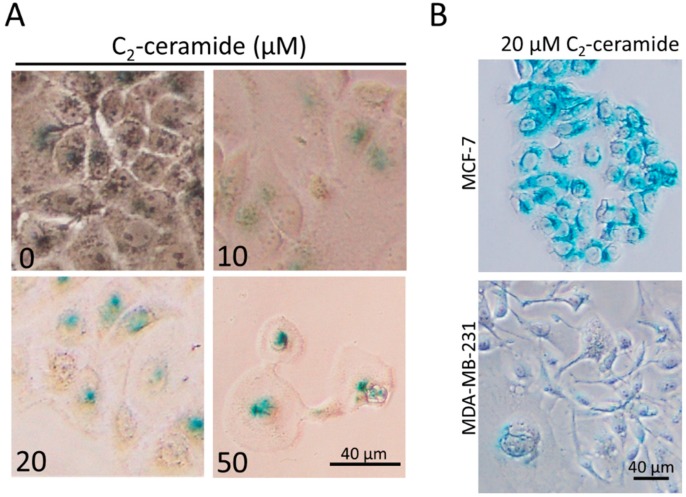
The detection of senescence-like phenotype using SA-β-gal staining. (**A**) MCF-7 cells were treated with the indicated doses of C_2_-ceramide for six days respectively. Afterward, the cells were glutaraldehyde-fixed and stained with the substrate X-gal (pH 6.0) for 24 h. Nought indicates the cells were treated with C_2_-ceramide-free solvent as vehicle control. (**B**) Breast cancer cells were cultured with 20 μM C_2_-ceramide respectively. The stained cells with green around the peri-nuclear regions were considered to be senescent cells.

Our preliminary results showed that numerous flattened and enlarged cells were observed in C_2_-ceramide-treated MCF-7, a hallmark of the senescence-like phenotype; accordingly, we sought to determine whether C_2_-ceramide could induce senescence-like phenotype in breast cancer cells. The acidic SA-β-gal staining was conducted for detecting the senescence at day six following C_2_-ceramide administration (Figure 2A). As shown in Figure 2A, the acidic SA-β-gal positive cells significantly increased in C_2_-ceramide-treated MCF-7. However, the same concentration (20 µM) of C_2_-ceramide induced senescence-like phenotype characteristics in MCF-7 rather than in MDA-MD-231 cells (Figure 2B).

### 2.3. C_2_-Ceramide Induced Apoptosis of MDA-MB-231 Cells

**Figure 3:**
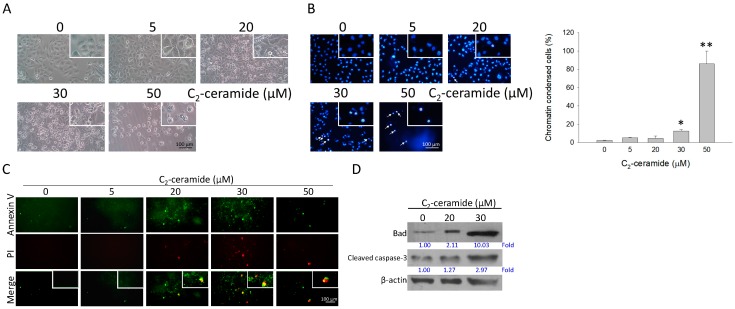
The detection of apoptosis in C_2_-ceramide-treated breast cancer cells. MDA-MB-231 cells were treated with the indicated concentrations of C_2_-ceramide (from 5 to 50 μM) for 24 h respectively. (**A**) The cells were observed using phase-contrast microscopy. (**B**) Chromatin condensation is shown, a hallmark of apoptosis induced by ceramide treatment. The white arrows indicate the chromatin condensation-positive cells. (**C**) The fluorescence microscope-based apoptosis assessment using annexin-V conjugated FITC and Propodium Iodide dual staining. (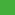 Annexin-V-positive, 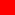 propidium iodide and 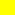 indicates the late stage of apoptotic cells). (**D**) The protein changes of pro-apoptotic Bad and cleavage of caspase-3 indicate an index of proteolytic activation. Nought indicates the cells were treated with C_2_-ceramide-free solvent as a vehicle control. β-actin as an internal control. Scale bar: 100 μM * *p* < 0.05, ** *p* < 0.01.

As shown in Figure 3A, the shrinkage and rounding of MDA-MB-231 cells were observed after 24 h treatments of C_2_-ceramide, especially at the 20 and 30 μM of C_2_-ceramide. Furthermore, ceramide treatments caused significant chromatin condensation, a hallmark of apoptosis in a dose-dependent manner (Figure 3B). The assay of fluorescence microscope-based Annexin V/Propidium Iodide staining further confirmed C_2_-ceramide induced apoptosis in MDA-MB-231. Besides the Annexin V positive cells, the dramatic decrease of cell number, and massive accumulation of Annexin V/PI-positive cells, a late stage of apoptosis was also observed by 50 μM of C_2_-ceramide treatments, indicating the susceptibility of MDA-MB-231 cells to higher concentrations (50 μM) of C_2_-ceramide. The results of Western blotting reveal upregulation of pro-apoptotic Bcl-2 protein Bad and the proteolytic activation of caspase-3 (cleaved caspase-3) following ceramide treatments (Figure 3D).

### 2.4. Expression Modulation of SA-Genes Was Modulated by C_2_-Ceramide

**Figure 4:**
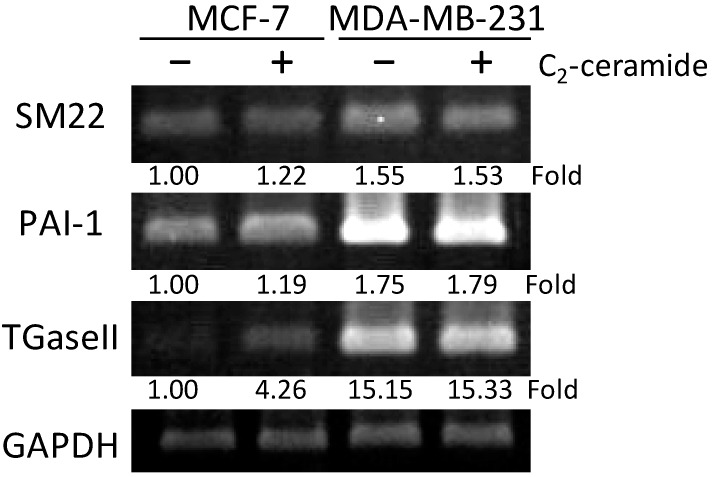
C_2_-ceramide-modulated RNA expression of senescence-associated genes in breast cancer cells. The two breast cancer MCF-7 and MDA-MB-231 cell lines treated with 20 μM C_2_-ceramide for 24 h respectively. SA-genes PAI-1 and TGaseII expression levels increased in MCF-7 cells but not in MDA-MB-231 cells. Glyceraldehyde-3-phosphate dehydrogenase (GAPDH) as an internal control. All fold changes were normalized by the level of internal control.

While senescence occurred, SA factors were activated to promote the senescence process. Thus, to further investigate the effect of C_2_-ceramide in inducing SA factor regulation, RT-PCR was performed to evaluate the gene expression of SA-genes. As shown in Figure 4, it was found that the mRNA levels of SA-genes of SM22 were not altered by C_2_-ceramide treatment. However, *PAI-I* and *TGase II* were upregulated 1.46-fold and 5.22-fold respectively following 20 µM C_2_-ceramide-treated MCF-7 for 24 h. In contrast, there was no significant alteration of SA-gene found in C_2_-ceramide-treated MD-MBA-231 cells. The results suggest that C_2_-ceramide induced a senescence-related signaling pathway in MCF-7 cells, rather than in MDA-MB-231 cells.

### 2.5. The Regulation of Senescence- and Pro-Apoptotic Factors in C_2_-Ceramide-Created Breast Cancer Cells

**Figure 5:**
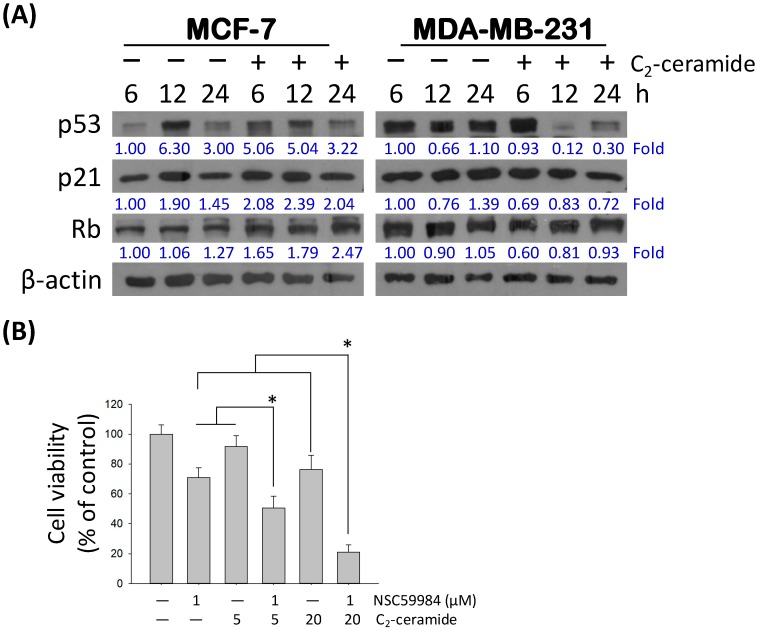
C_2_-ceramide-induced apoptosis-related and senescence-related signaling pathway. (**A**) The expression changes of SA- and pro-apoptotic proteins in C_2_-ceramide-treated breast cancer cells. The two breast cancer cell lines were treated with 20 μM of C_2_-ceramide for 6, 12, and 24 h respectively. β-actin as an internal control. All fold changes were normalized by the level of internal control. (**B**) The investigation of *p*53 activator NSC59984 (1 µM) co-treated with C_2_-ceramide (5 and 20 µM respectively) 24 h for cell viability analysis. * *p* < 0.05.

The regulatory effect of C_2_-ceramide in inducing senescence- and pro-apoptosis factors in MCF-7 and MDA-MB-231 cells was further investigated. We found that C_2_-ceramide induced a rapid increase of *p*53 5.06-fold at the early time point of 6 h and then decreased to basal expression after 24 h in MCF-7; however, C_2_-ceramide significantly suppressed the protein expression of mutant *p*53 from 12 h to 24 h after treatment in MDA-MB-231 cells (Figure 5A, first line), indicating that C_2_-ceramide induced MDA-MB-231 cell apoptosis by reducing pro-proliferating mutant *p*53. In contrast, compared with a slight increase of p21, C_2_-ceramide significantly up-regulated Rb protein expression in MCF-7 cells up to 2.47-fold in a time-dependent manner, whereas there was no obvious change compared with that in MDA-MB-231 cells (Figure 5A). p21 and Rb have been reported as senescence-related markers [31], indicating that C_2_-ceramide specifically induced senescence marker p21 and Rb protein activation to promote the senescence of MCF-7 cells. To further characterize the importance of *p*53 in C_2_-ceramide-induced MCF-7 apoptosis, the *p*53 activator NSC59984 was used. Through single or co-treatment of NSC59984 and C_2_-ceramide, we found that NSC59984 significantly enhanced the cytotoxicity by increasing cell death by 40% to 60% in C_2_-ceramide-treated MCF-7 cells (Figure 5B), suggesting that the activation of wild-type *p*53 resensitizes MCF-7 cells toward C_2_-ceramide-induced death, and that the effect of *p*53 activation was more dominant than Rb-mediated senescence.

## 3. Discussion

Our previous studies have revealed the role of C_2_-ceramide as a promising strategy for lung cancer therapies [26,32,33,34]. Ceramide has been validated as safe toward normal cells and for its selective cytotoxicity toward cancer cells. For example, C_2_-ceramide induced extremely low cytotoxicity in human dermal neonatal fibroblast (HDNF) cells with 66.5 μM of IC_50_ (24 h) dosage [35], and it was even more effective in the normal lung cell lines Beas-2B and MRC-5 cells [36]. In the current study, we provided evidence regarding how C_2_-ceramide induced apoptotic cell death in two breast cancer cell lines. We found that C_2_-ceramide induced discrepant cytotoxicity in breast cancer cell lines MCF-7 and MDA-MB-231, with the IC_50_ of 27.13 µM in MCF-7 and 4 µM in MDA-MB-231 respectively (Figure 1). Interestingly, C_2_-ceramide induced an extra effect of senescence phenotype in MCF-7 rather than MDA-MB-231 (Figure 2). Thus, this might be the main reason why MCF-7 was more resistant to C_2_-ceramide-induced cytotoxicity than MDA-MB-231. Twenty µM of C_2_-ceramide induced significant senescence-like phenotype in MCF-7 with the rise of acid ß-gal positive staining cells, whereas the same dose of C_2_-ceramide induced obvious apoptotic features of Annexin-V positive staining in MDA-MB-231 cells (Figure 3). Moreover, we found that MDA-MB-231 bear mutant *p*53 was also involved in C_2_-ceramide-induced apoptosis, suggesting that the reduction of mutant *p*53 was involved in the increased susceptibility of MDA-MB-231 to C_2_-ceramide treatment.

Senescence plays the role of tumor suppressor against cancer cell proliferation. Under normal conditions, the induction of senescence is reported as being part of *p*53-induced apoptosis and acts as a tumor suppressor-like signaling. The microRNA miR-34a is found to be one of the upstream regulators of senescence in the *p*53-related pathway [37,38]. The overexpression of miR-34a causes an increase of senescence and a reduction of cell proliferation of the human colon cancer HCT116 cell line via downregulation of E2F-related protein and cell cycle regulators; however, reports increasingly support a different role of senescence in promoting tumor cell resistance to therapeutic drug-induced cellular stress and cytotoxicity. An opposite role of senescence is reported as being an oncogenic protector that prevents the cell from cell death regulation in a cellular process. The senescent cell is found to escape from therapy drug-induced cell cycle arrest and sustained cell death by over-expressing Cdc2/Cdk1 kinase activity and prolonging cell cycle arrest. Survivin is reported to be an important regulator that is phosphorylated and cooperates with Cdc2/Cdk1 to inhibit apoptosis signaling, and the reverse results are shown to induce apoptosis [39].

Besides, PAI-1 (plasminogen activator inhibitor-1) has been considered as a marker and a mediator of senescence [40], and as an upstream regulator of *p*53 and down-stream of insulin-like growth factor binding protein-3, and the inhibition or any functional genetic mutation of PAI-1 would result in the resistance of senescence [41]. Another senescence marker, Transglutaminase II (TGaseII), is also reported to be an aging-related senescent cell marker [42]. TGaseII knockout mice present a markedly attenuated endothelial-dependent vasodilation (EDV) than that of wild-type mice, with regularly well-arranged elastic laminae of the tunica media [42]. In this case, C_2_-ceramide may induce similar signaling transduction to initiate the senescence pathway by increasing the expression of SA-associated genes PAI-1 and TGaseII (Figure 4). Strong evidence shows that 20 µM of C_2_-ceramide significantly induced cell senescence in MCF-7 cells but not MDA-MB-231 cells, and this might provide cell protection against external stress and cell death, thus possibly explaining why MCF-7 appears more resistant to C_2_-ceramide-induced cytotoxicity.

Accumulated evidence supports the existence of at least two different signaling pathways to trigger cell senescence: the *p*53-dependent and Rb-dependent pathways [43]. Moreover, Rb-dependent senescence appears to be potentially reversible after inactivating Rb expression [43]. In our case, it showed that C_2_-ceramide activated wild-type *p*53 of MCF-7 cells rapidly at the early time point of 6 h, whereas the effect decreased from 12 to 24 h dramatically. Instead of *p*53, Rb protein sustained increase from 6 to 24 h in MCF-7 cells. We suggest that C_2_-ceramide induced a reversible Rb-dominant senescence-like phenotype (SLP) in MCF-7.

**Figure 6:**
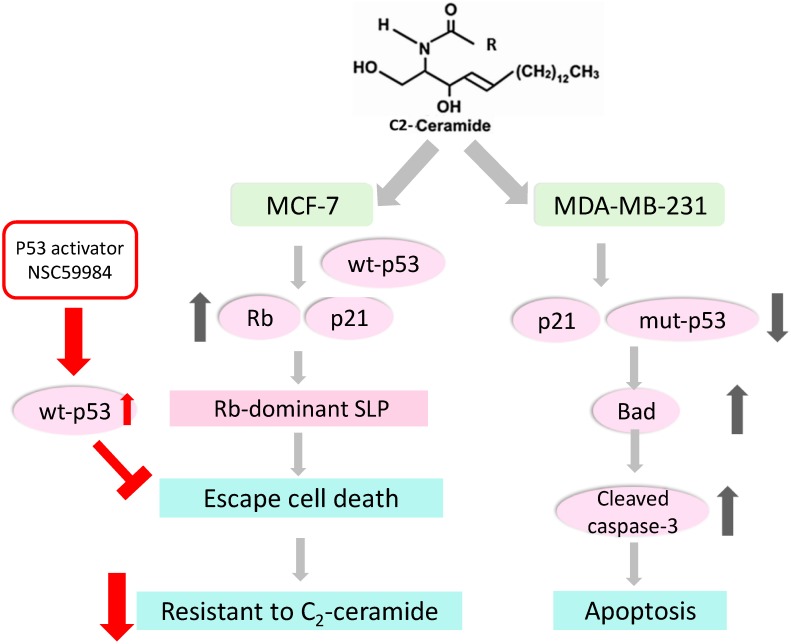
A proposed mechanism whereby breast cancer cells escape C_2_-ceramide-induced apoptosis through modulating senesce-like phenotype. Exogenous C_2_-ceramide inhibits growth arrest and induces apoptosis in breast cancer MDA-MB-231 cells through down-regulating the expression of mutant *p*53 while up-regulating pro-apoptosis pathways, including the expression of Bax and Bad and the proteolytic activation of caspase-3. In contrast, C_2_-ceramide treatments that favor the induction of senescence-like phenotype might occur through the activation of Rb rather than the activation of *p*53-signaling of senescence. Rb-mediated senescence-like phenotype (SLP) might be reversible and confer more resistance of breast cancer MCF-7 cells to C_2_-ceramide. In contrast, the activation of wild type *p*53 using a *p*53 activator resensitizes MCF-7 cells to C_2_-ceramide, suggesting the critical role of wild type *p*53 in C_2_-ceramide-induced death in breast cancer cells. Therefore, the proposed model suggests that some cancer cells escape apoptosis induction through modulating senescence-like phenotype, whereas the *p*53 activation can overcome the chemoresistance and should be a promising strategy for treating cancer cells which favor stress-induced SLP. (↑, increase or upregulation; ↓, decrease or downregulation; T, attenuation or blockade).

Most importantly, senescent cells are reported to be more inactive and resistant to induction of cell death, suggesting that cancer cells might escape death through modulating reversible SLP. For example, senescent cells harbored a low expression of Bcl-2, a marker of the pro-survival protein showing resistance to H_2_O_2_-induced cell death [44]. It seems that the Rb-dependent reversible senescence activated by C_2_-ceramide might contribute to the finding as to why MCF-7 is more resistant to certain therapeutic drug treatments (Figure 6).

It has been reported that mutant *p*53 acts in a dominant-negative role in cancer progression and may provide cell protection. Although *p*53 plays a tumor-suppressor role against tumorigenesis, over 50% of breast cancer patients and 80% of triple-negative breast cancer (TNBC) patients bear mutant *p*53, demonstrating that mutant *p*53 plays a vital role in breast cancer progression [45]. The cumulating data show that mutant *p*53 exhibits loss of tumor suppressor activity and gains the function of promoting cancer survival. In the current study, an opposite type of *p*53 was present in the two breast cancer cell lines, MCF-7 cells with wild type *p*53, and MDA-MB-231 cells with mutant *p*53. We found that C_2_-ceramide induced great inhibition of mutant *p*53 expression in MDA-MB-231 cells, and a slight increase of wild type *p*53 expression in MCF-7 cells. We provided further evidence regarding that when combined with *p*53 activator NSC59984, the cytotoxicity of C_2_-ceramide in MCF-7 cells was significantly enhanced, indicating that the activation of *p*53 and its downstream signaling and the anti-cancer effect induced by C_2_-ceramide were two individual actions, and so the effect of combined treatment was evident (Figure 5B). It also suggests that C_2_-ceramide could induce greater cytotoxicity in MCF-7 once the *p*53-related pathway was activated, and cells would no longer escape from Rb-mediated SLP to recover damage induced by C_2_-ceramide.

The effect of exogenous C_2_-ceramide in the endogenous long-chain sphingolipid pathway was also investigated; accordingly, exogenous ceramide analogs was determined to have great opportunity to induce or modify the endogenous ceramide species and associated signaling while activating apoptosis-related signaling. This shows that synthetic C_6_-ceramide in turn activated the generation of cellular C_16_-ceramide concentration as well as the synthetic ceramide analogs HPL-39N and HPL-1R36N induced in tumor cells. M Blaess et al. suggest that these synthetic ceramides induce a significant effect on the expression of a gene involved in cell cycle, cell growth and cell death, which contributes to apoptosis induction [46]. Moreover, evidence is shown that these exogenous ceramides would be partially incorporated into the newly synthesized long-chain ceramides via the deacylation-reacylation cycle, and then cause the regulation of growth inhibition, cell cycle arrest, and modulation of telomerase activity [47]. In this case, we suggest that the exogenous C_2_-ceramide might act in the same way while inducing breast cancer cell apoptosis due to the phenomenon of C_2_-ceramide in inducing cancer cell growth inhibition, as cycle arrest has been well-proven [48,49].

Although C_2_-ceramide induced great cytotoxicity in MCF-7 and MDA-MB-231 cells with different mechanisms, there is some concern about a safety issue. Recently, it has been reported that when compared with normal breast stem cells, Type I human breast epithelial cell (HBEC), C_2_-ceramide induces slight cytotoxicity in Type II HBECs [50], suggesting that the use of C_2_-ceramide needs to be optimized to reduce such cytotoxicity in normal breast cells. Our previous study provides evidence regarding the usage of C_2_-ceramide in combination with clinical drugs for enhanced treatment efficacy [36]. Combining sub-lethal C_2_-ceramide and Chloroquine (CQ) greatly enhances the cytotoxicity in NSCLC cells but shows extremely low cytotoxicity in normal lung cells, suggesting that it might exhibit the same alternative use of C_2_-ceramide in treating breast cancer cells with less burden fornormal breast tissue or epithelial cells.

Taken together, our data support that C_2_-ceramide induced apoptosis in two breast cancer cell lines, including MCF-7 and MDA-MB-231 cells. C_2_-ceramide induced high cytotoxicity in MDA-MB-231 cells by targeting mutant *p*53 expression. Conversely, C_2_-ceramide triggered senescence-signaling transduction in MCF-7 cells and resulted in MCF-7 cells escaping from C_2_-ceramide-induced apoptosis. The senescence phenomenon provides a reliable explanation of why certain breast cancer cells show strong chemoresistance against therapeutic drugs during the treatment process (Figure 6). The current study also provided an insight showing that the alternative pharmacotherapy of C_2_-ceramide should be provided to patients harboring genetic mutations such as *p*53 mutation.

## 4. Materials and Methods

### 4.1. Preparation of C_2_-Ceramide

Short carbon-chain C_2_-ceramide (N-Acetyl-D -sphingosine) was purchased from Sigma (#A7191, St. Louis, MO, USA) and was dissolved in DMSO (Sigma-Aldrich, Munich, Bavaria, Germany). C_2_-ceramide were aliquoted and stocked at −20 °C.

### 4.2. Reagents

DMEM/F-12 Medium, the antibiotics streptomycin/penicillin G and fetal bovine serum (FBS) were purchased from Gibco (Gaithersburg, MD, USA). The antibodies against the following proteins Bad (#sc8044) were purchased from Santa Cruz Biotechnology (Santa Cruz, CA, USA). Bax (#GTX109683) was purchased from GeneTex (Irvine, CA, USA). Caspase 3 (#IMG144A) was purchased from IMGENEX (San Diego, CA, USA). p21 (#2947P) was purchased from Cell Signaling Technology (Beverly, MA, USA). *p*53 (#1026-1) was purchased from Epitomics (Epitomics, Burlingame, CA, USA). β-actin (#BD612656) was purchased from BD Biosciences (Franklin Lakes, NJ, USA). Horseradish peroxidase (HRP)-conjugated secondary antibodies (#20102 for goat anti-mouse IgG, #20202 for goat anti-rabbit IgG) was purchased from Leadgene Biomedical Inc. Tainan, Taiwan. The Annexin V-fluorescein isothiocyanate (FITC) kit was purchased from Strong Biotech Co. (#AVK050, Taipei, Taiwan).

### 4.3. Cell Cultures

The human breast cancer cell lines MCF-7 (wild type *p*53) and MDA-MB-231 (mutant *p*53) (American Type Culture Collection, Manassas, VA, USA) were maintained in the medium DMEM and F12 supplemented (1:1 ratio) with 10% fetal bovine serum (FBS; Gibco BRL, Grand Island, NY, USA) and 0.5% streptomycin/penicillin (Mediatech, Inc., Herndon, VA, USA) at 37 °C with 5% CO_2_.

### 4.4. Cell Proliferation Assessment

The cellular proliferation was assessed using a WST-1 assay. Briefly, a total of 1 × 10^3^/well and culture cells in 96-well plates in a final volume of 100 µL/well medium were treated with the indicated concentrations of C_2_-ceramide for 24 h respectively. BPIQ, a synthetic analog of camptothecin as a positive control [51], was used. Ten μL/well WST-1 reagent kit (Takara Biochem., Tokyo, Japan) was added and incubated for 30 min. The absorbance of the samples against a background control as blank was measured by a microplate reader (Multiskan Ascent 354, Thermo Fisher Scientific, Rockford, IL, USA) at 450 nm.

### 4.5. Apoptosis Assessment

Annexin staining was performed for detecting apoptosis [36]. The cells were treated with the indicated concentrations of C_2_-ceramide for 24 h respectively. Afterward, the cells were stained with 10 μg/mL of Annexin V-FITC, and the cells were observed by a fluorescence microscope (TE2000-U; Nikon, Tokyo, Japan).

### 4.6. Senescence-Associated β-Galactosidase (SA-β-gal) Staining

The staining of SA-β-gal was used for detecting the cellular senescence [52]. The protocol used in the study was according to the previously published one with minor modifications [48]. In brief, cells were treated with C_2_-ceramide for six days. Afterward, the cells were washed twice with phosphate-buffered saline (PBS) and glutaraldehyde-fixed, then 1 mg/mL of 5-bromo-4-chloro-3-indoyl-β-galactoside was added (X-gal which was dissolved in a solvent composed of dimethylformamide, five mM potassium ferrocyanide, 150 mM NaCl, 40 mM citric acid and sodium phosphate, and 2 mM MgCl_2_, pH6.0) for 24 h. The cells were then washed with PBS, and the β-gal (green color) stained cells were determined. Magnification 200×.

### 4.7. Reverse Transcription-qPCR Assessment

1 × 10^6^ cells were treated with C_2_-ceramide for 24 h. The harvested cells were lysed with RNA extraction kit EasyBlue™ (iNtRON, Seoul, Korea) according to the instructions of the manufacturer. The quantity of total RNA was measured using OD_260/280 nm,_ and then the quality was checked using electrophoresis on a 1.0% agarose gel with 2.2 M formaldehyde. The information of primer sequences is presented in Table 1. The primers of senescence-associated genes were designed based on the study previously published [48]. The PCR products were electrophoresis-resolved and densitometric-analyzed using Gel-Pro v.4.0 software (Media Cybernetics, Silver Spring, MD, USA).

### 4.8. Western Blotting Assay

20 μg of protein lysates were resolved using the 10% SDS-polyacrylamide gel and then transferred to a nitrocellulose membrane. The transferred NC membranes were blocked with 5% nonfat milk. Subsequently, the membranes were reacted with primary antibodies and the corresponding secondary antibodies sequentially. The signals of specific protein were detected using a chemiluminescence detection kit (ECL™, Amersham, Piscataway, NJ, USA).

### 4.9. Assessment of p53 Activator

The survival rates of MCF-7 cells incubated either with C_2_-ceramide single treatment or *p*53 activator co-treatment were determined using the MTS Cell Proliferation Assay Kit (Promega, Madison, WI, USA). Briefly, 1 × 10^3^ cells were seeded and pretreated with the indicated concentrations of *p*53 activator NSC59984 (Cat No. B6045 APExBIO, Houston, TX, USA) prior to the treatment of indicated concentrations of C_2_-ceramide (Sigma-Aldrich) for 24 h. Afterward, the MTS solution was added to the cells and further incubated for 1 h at 37 °C according to the manual of the manufacturer. Afterward, the cell viability was determined using the absorbance at 490 nm using a microreader (Biorad, Model 550, Hercules, CA USA).

### 4.10. Statistical Analysis

All data were presented as mean ± S.D. Differences between controls and treatment groups were analyzed by Student’s *t*-test.

## Figures and Tables

**Table 1:** Primer pairs used in the study.

Target Gene	Forward Primer (5′-3′)	Reverse Primer (5′-3′)	Size (bp)
GAPDH	CGTCTTCACCATGGAGA	CGGCCATCACGCCCACAGTTT	310
PAI-1	GTGTTTCAGCAGGTGGCGC	CCGGAACAGCCTGAAGAAGTG	310
SM22	TGGCGTGATTCTGAGCAA	CTGCCAAGCTGCCCAAGG	534
TGase II	CTCGTGGAGCCAGTTATCAACAGCTAC	TCTCGAAGTTCACCACCAGCTTGTG	310

## References

[1] Akram M., Iqbal M., Daniyal M., Khan A.U. (2017). Awareness and current knowledge of breast cancer. Biol. Res..

[2] Hayflick L. (1965). The limited in vitro lifetime of human diploid cell strains. Exp. Cell Res..

[3] Gewirtz D.A., Holt S.E., Elmore L.W. (2008). Accelerated senescence: An emerging role in tumor cell response to chemotherapy and radiation. Biochem. Pharm..

[4] Mathon N.F., Lloyd A.C. (2001). Milestones in cell division: Cell senescence and cancer. Nat. Rev. Cancer.

[5] Choudhury A.R., Ju Z., Djojosubroto M.W., Schienke A., Lechel A., Schaetzlein S., Jiang H., Stepczynska A., Wang C., Buer J. (2007). Cdkn1a deletion improves stem cell function and lifespan of mice with dysfunctional telomeres without accelerating cancer formation. Nat. Genet..

[6] D’Adda di Fagagna F., Reaper P.M., Clay-Farrace L., Fiegler H., Carr P., Von Zglinicki T., Saretzki G., Carter N.P., Jackson S.P. (2003). A DNA damage checkpoint response in telomere-initiated senescence. Nature.

[7] Campisi J. (2001). Cellular senescence as a tumor-suppressor mechanism. Trends Cell Biol..

[8] Serrano M., Lin A.W., McCurrach M.E., Beach D., Lowe S.W. (1997). Oncogenic ras provokes premature cell senescence associated with accumulation of p53 and p16INK4a. Cell.

[9] Braig M., Lee S., Loddenkemper C., Rudolph C., Peters A.H., Schlegelberger B., Stein H., Dorken B., Jenuwein T., Schmitt C.A. (2005). Oncogene-induced senescence as an initial barrier in lymphoma development. Nature.

[10] Zhuang D., Mannava S., Grachtchouk V., Tang W.H., Patil S., Wawrzyniak J.A., Berman A.E., Giordano T.J., Prochownik E.V., Soengas M.S. (2008). C-MYC overexpression is required for continuous suppression of oncogene-induced senescence in melanoma cells. Oncogene.

[11] Bartkova J., Rezaei N., Liontos M., Karakaidos P., Kletsas D., Issaeva N., Vassiliou L.V., Kolettas E., Niforou K., Zoumpourlis V.C. (2006). Oncogene-induced senescence is part of the tumorigenesis barrier imposed by DNA damage checkpoints. Nature.

[12] Al-Hajj M., Wicha M.S., Benito-Hernandez A., Morrison S.J., Clarke M.F. (2003). Prospective identification of tumorigenic breast cancer cells. Proc. Natl. Acad. Sci. USA.

[13] Goodell M.A., McKinney-Freeman S., Camargo F.D. (2005). Isolation and characterization of side population cells. Methods Mol. Biol..

[14] Ho M.M., Ng A.V., Lam S., Hung J.Y. (2007). Side population in human lung cancer cell lines and tumors is enriched with stem-like cancer cells. Cancer Res..

[15] Sionov R.V., Haupt Y. (1999). The cellular response to p53: The decision between life and death. Oncogene.

[16] Lacroix M., Toillon R.A., Leclercq G. (2006). p53 and breast cancer, an update. Endocr. Relat. Cancer.

[17] Prives C., Hall P.A. (1999). The p53 pathway. J. Pathol..

[18] He C., Li L., Guan X., Xiong L., Miao X. (2017). Mutant p53 Gain of Function and Chemoresistance: The Role of Mutant p53 in Response to Clinical Chemotherapy. Chemotherapy.

[19] Hientz K., Mohr A., Bhakta-Guha D., Efferth T. (2017). The role of p53 in cancer drug resistance and targeted chemotherapy. Oncotarget.

[20] Knappskog S., Lonning P.E. (2012). P53 and its molecular basis to chemoresistance in breast cancer. Expert Opin. Ther. Targets.

[21] Zhang S., Zhou L., Hong B., van den Heuvel A.P.J., Prabhu V.V., Warfel N.A., Kline C.L.B., Dicker D.T., Kopelovich L., El-Deiry W.S. (2015). Small-molecule NSC59984 restores p53 pathway signaling and antitumor effects against colorectal cancer via p73 activation and degradation of mutant p53. Cancer Res..

[22] Yan W., Zhang Y., Zhang J., Liu S., Cho S.J., Chen X. (2011). Mutant p53 protein is targeted by arsenic for degradation and plays a role in arsenic-mediated growth suppression. J. Biol. Chem..

[23] Gordon R.R., Nelson P.S. (2012). Cellular senescence and cancer chemotherapy resistance. Drug Resist. Updates.

[24] Skolová B., Hudská K.R., Pullmannová P., Kováčik A., Palát K., Roh J., Fleddermann J., Estrela-Lopis I., Vávrová K. (2014). Different phase behavior and packing of ceramides with long (C16) and very long (C24) acyls in model membranes: Infrared spectroscopy using deuterated lipids. J. Phys. Chem. B.

[25] Goldkorn T., Chung S., Filosto S. (2013). Lung cancer and lung injury: The dual role of ceramide. Sphingolipids in Disease.

[26] Flowers M., Fabriás G., Delgado A., Casas J., Abad J.L., Cabot M.C. (2012). C6-ceramide and targeted inhibition of acid ceramidase induce synergistic decreases in breast cancer cell growth. Breast Cancer Res. Treat..

[27] Demarchi F., Bertoli C., Greer P., Schneider C. (2005). Ceramide triggers an NF-κB-dependent survival pathway through calpain. Cell Death Differ..

[28] Xu L., Deng X. (2006). Suppression of cancer cell migration and invasion by protein phosphatase 2A through dephosphorylation of μ-and m-calpains. J. Biol. Chem..

[29] Lin I.L., Chou H.L., Lee J.C., Chen F.W., Fong Y., Chang W.C., Huang H.W., Wu C.Y., Chang W.T., Wang H.D. (2014). The antiproliferative effect of C2-ceramide on lung cancer cells through apoptosis by inhibiting Akt and NFkappaB. Cancer Cell Int..

[30] Chang Y.C., Fong Y., Tsai E.M., Chang Y.G., Chou H.L., Wu C.Y., Teng Y.N., Liu T.C., Yuan S.S., Chiu C.C. (2018). Exogenous C (8)-Ceramide Induces Apoptosis by Overproduction of ROS and the Switch of Superoxide Dismutases SOD1 to SOD2 in Human Lung Cancer Cells. Int. J. Mol. Sci..

[31] Jiang C., Liu G., Luckhardt T., Antony V., Zhou Y., Carter A.B., Thannickal V.J., Liu R.M. (2017). Serpine 1 induces alveolar type II cell senescence through activating p53-p21-Rb pathway in fibrotic lung disease. Aging Cell.

[32] Morad S.A., Messner M.C., Levin J.C., Abdelmageed N., Park H., Merrill A.H., Cabot M.C. (2013). Potential role of acid ceramidase in conversion of cytostatic to cytotoxic end-point in pancreatic cancer cells. Cancer Chemother. Pharm..

[33] Huang H., Zhang Y., Liu X., Li Z., Xu W., He S., Huang Y., Zhang H. (2011). Acid sphingomyelinase contributes to evodiamine-induced apoptosis in human gastric cancer SGC-7901 cells. DNA Cell Biol..

[34] Fabrias G., Bedia C., Casas J., Abad J.L., Delgado A. (2011). Ceramidases in hematological malignancies: Senseless or neglected target?. Anti Cancer Agents Med. Chem..

[35] Kang J.H., Garg H., Sigano D.M., Francella N., Blumenthal R., Marquez V.E. (2009). Ceramides: Branched alkyl chains in the sphingolipid siblings of diacylglycerol improve biological potency. Bioorg. Med. Chem..

[36] Chou H.L., Lin Y.H., Liu W., Wu C.Y., Li R.N., Huang H.W., Chou C.H., Chiou S.J., Chiu C.C. (2019). Combination Therapy of Chloroquine and C2-Ceramide Enhances Cytotoxicity in Lung Cancer H460 and H1299 Cells. Cancers.

[37] Chang T.C., Wentzel E.A., Kent O.A., Ramachandran K., Mullendore M., Lee K.H., Feldmann G., Yamakuchi M., Ferlito M., Lowenstein C.J. (2007). Transactivation of miR-34a by p53 broadly influences gene expression and promotes apoptosis. Mol. Cell.

[38] He L., He X., Lim L.P., de Stanchina E., Xuan Z., Liang Y., Xue W., Zender L., Magnus J., Ridzon D. (2007). A microRNA component of the p53 tumour suppressor network. Nature.

[39] Wang Q., Wu P.C., Roberson R.S., Luk B.V., Ivanova I., Chu E., Wu D.Y. (2011). Survivin and escaping in therapy-induced cellular senescence. Int. J. Cancer.

[40] Vaughan D.E., Rai R., Khan S.S., Eren M., Ghosh A.K. (2017). Plasminogen Activator Inhibitor-1 Is a Marker and a Mediator of Senescence. Arter. Thromb. Vasc. Biol..

[41] Eren M., Boe A.E., Klyachko E.A., Vaughan D.E. (2014). Role of plasminogen activator inhibitor-1 in senescence and aging. Semin. Thromb. Hemost..

[42] Armstrong D.M.F., Sikka G., Armstrong A.D.C., Saad K.R., Freitas W.R., Berkowitz D.E., Fagundes D.J., Santhanam L., Taha M.O. (2018). Knockdown of transglutaminase-2 prevents early age-induced vascular changes in mice1. Acta Cir. Bras..

[43] Chakradeo S., Elmore L.W., Gewirtz D.A. (2016). Is Senescence Reversible?. Curr. Drug Targets.

[44] Sasaki M., Kumazaki T., Takano H., Nishiyama M., Mitsui Y. (2001). Senescent cells are resistant to death despite low Bcl-2 level. Mech. Ageing Dev..

[45] Synnott N.C., Bauer M.R., Madden S., Murray A., Klinger R., O’Donovan N., O’Connor D., Gallagher W.M., Crown J., Fersht A.R. (2018). Mutant p53 as a therapeutic target for the treatment of triple-negative breast cancer: Preclinical investigation with the anti-p53 drug, PK11007. Cancer Lett..

[46] Blaess M., Le H.P., Claus R.A., Kohl M., Deigner H.P. (2015). Stereospecific induction of apoptosis in tumor cells via endogenous C16-ceramide and distinct transcripts. Cell Death Discov..

[47] Ogretmen B., Pettus B.J., Rossi M.J., Wood R., Usta J., Szulc Z., Bielawska A., Obeid L.M., Hannun Y.A. (2002). Biochemical mechanisms of the generation of endogenous long chain ceramide in response to exogenous short chain ceramide in the A549 human lung adenocarcinoma cell line. Role for endogenous ceramide in mediating the action of exogenous ceramide. J. Biol. Chem..

[48] Chen J.Y., Hwang C.C., Chen W.Y., Lee J.C., Fu T.F., Fang K., Chu Y.C., Huang Y.L., Lin J.C., Tsai W.H. (2010). Additive effects of C (2)-ceramide on paclitaxel-induced premature senescence of human lung cancer cells. Life Sci..

[49] Spyridopoulos I., Mayer P., Shook K.S., Axel D.I., Viebahn R., Karsch K.R. (2001). Loss of cyclin A and G1-cell cycle arrest are a prerequisite of ceramide-induced toxicity in human arterial endothelial cells. Cardiovasc. Res..

[50] Ahn E.H., Yang H., Hsieh C.Y., Sun W., Chang C.C., Schroeder J.J. (2019). Evaluation of chemotherapeutic and cancer-protective properties of sphingosine and C2-ceramide in a human breast stem cell derived carcinogenesis model. Int. J. Oncol..

[51] Chiu C.C., Chou H.L., Chen B.H., Chang K.F., Tseng C.H., Fong Y., Fu T.F., Chang H.W., Wu C.Y., Tsai E.M. (2015). BPIQ, a novel synthetic quinoline derivative, inhibits growth and induces mitochondrial apoptosis of lung cancer cells in vitro and in zebrafish xenograft model. BMC Cancer.

[52] Dimri G.P., Lee X., Basile G., Acosta M., Scott G., Roskelley C., Medrano E.E., Linskens M., Rubelj I., Pereira-Smith O. (1995). A biomarker that identifies senescent human cells in culture and in aging skin in vivo. Proc. Natl. Acad. Sci. USA.

